# Reachable sets of differential inclusions: New DI solver, parallel version, interdisciplinary applications

**DOI:** 10.1016/j.mex.2021.101439

**Published:** 2021-07-02

**Authors:** Stanislaw Raczynski

**Affiliations:** Universidad Panamericana, Facultad de Ingeniería, Augusto Rodin 498, Ciudad de México, 03920, México

**Keywords:** Differential inclusions, Reachable set, Optimal control, uncertainty

## Abstract

The new version of the differential inclusion solver is presented. The solver calculates the reachable sets of differential inclusions. The new features are discussed, compared to the original version published in 2002. A short overview of differential inclusions and their relation to control systems is given. The new, parallel algorithm of the solver is presented. The concept of functional sensitivity is discussed, and the applications of the differential inclusion solver are discussed.

• The new, parallel version of the algorithm for reachable set calculation is described.
• Uncertainty treatment by differential inclusions is discussed.
• A new concept of functional sensitivity is proposed.

## Graphical abstract

**Figure:**
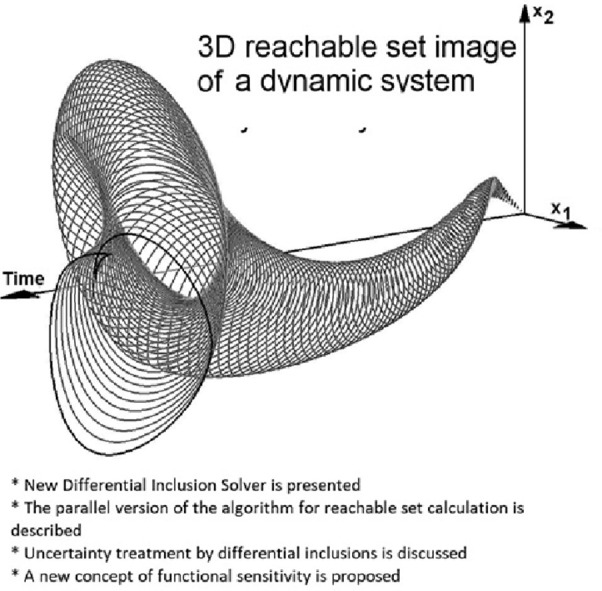

Specifications table

**Table:** 

Subject area:	Computer science
More specific subject area:	Algorithm for reachable set calculation for differential inclusions. New, parallel version of the solver is presented
Method name:	Differential Inclusion Solver
Name and reference of original method:	Raczynski S. (2002) Differential Inclusion Solver. Conference paper: International Conference on Grand Challenges for Modeling and Simulation, The Society for Modeling and Simulation Int., San Antonio TX.
Resource availability:	*If applicable, include links to resources necessary to reproduce the method (e.g. data, software, hardware, reagent)*

## Introduction

The first version of the differential inclusion solver (DI Solver) was presented two decades ago, see Raczynski [17]. We present this paper because the publication of 2002 was only a conference paper and has been unnoted by many researches. The other reason is that during the last two decades important improvements to the algorithm and related software have been done. Also, what is new in the present article, is the application of the DI solver to new concept of functional sensitivity analysis, described in the following sections.

Though the main idea of the solver remains the same, these modifications and new implementation deserve to be published and made known to eventual users. A more detailed definitions and specifications of the differential inclusions (DI) and attainable or reachable sets (RS) will be given in the following section. Here, let us give the general concepts.

First of all, note that the main idea of the software is to scan the boundary, and not the interior of the reachable set. Our point is that to explore the interior of the reachable set is an error. The reasons are as follows.

1. Obviously, the area of the boundary of the reachable set grows with the square of the model dimension, while the volume of its interior increases as the cube of the dimension.
2. The properties of the boundary of the are perfectly known for many decades. This makes the RS calculation easier because the algorithm is based on already proved and well documented methods of the optimal control theory (see [9], [16]).

The application of the optimal control methods for RS calculation has been re-invented several years after the original publication of 2002, see, for example Baier et al.

There are some other approaches to the RS determination, like, for example Girard et al. [8]. However, the method is limited to time-invariant linear systems. There are many other attempts to estimate reachable sets. For example, Matviychuk proposes external ellipsoidal approximation method. We will not comment here other numerous publications on this topic because nearly all of them propose little efficient methods and have been published after 2002, when the problem has been already solved. This remark does not refer to works that treat with extensions of the problem and non-classis cases, like systems with time-delay or fuzzy logic elements. For more references related to reachable set calculations and an overview, consult Filippova [5]. Consult also Bayer [3], Matviychuk [11].

## Differential inclusions

Let us recall the main concepts of differential inclusions. For a comprehensive overview of the topic consult Aubin and Cellina [1]. The early publications on DIs date from 1930s, see papers of Marchaud [10], Zaremba [27], Wazewski, [25], [26], Plis [14] and Turowicz [21], [22].

We delimit the definitions to the real Euclidean n-dimensional space *R^n^.* Some generalizations of differential inclusions and properties of the reachable sets in more abstract, Banach spaces can be found in the Journal of Mathematical Analysis and Applications, see Raczynski [18].

In the original Wazewski works on DIs, the field of directions in *R^n^*, defined by the multi-valued function *F(x,t)* of (1.1) is called *orientor field*. As the Wazewski's terminology is not commonly used in recent publications, we will call this field simply *F***,** when no relevant ambiguity may arise (do not confuse *F* with the field concept of the advanced algebra).

In the following, *I* is an interval of *R*.

We assume that the reader is familliar with the terms *almost everywhere, Lipshitz condition, absolutely continuous function* and *set-to-point distance*.

Let *X* and *Y* be two non-empty subsets of a metric space. The Hausdorff distance between *X* and *Y* is defined

$$d_{H}\left(X,Y\right)=\max\left\{\underset{x\in X}{\sup}\;\;\underset{y\in Y}{\inf}\;d\left(x,y\right),\;\underset{y\in Y}{\sup}\;\underset{x\in X}{\inf}\;d\left(x,y\right)\right\},$$

where sup represents the least upper bound, and d(*,*) is the distance between two points.

The Hausdorff distance permits to use the concept of continuity of set-valued functions. We say that a mapping from the real line to the space of closed subsets of *R^n^* is *continuous in Hausdorff sense* if it is continuous in the sense of the Hausdorff distance (in the topology induced by the Hausdorff distance).

Suppose that *f* is a real valued function *f: R^n^* → *R,* x_o_ ∈ *R^n,^ f(x_o_),* and *f* has a finite value at *x_o_*. The function *f* is *upper semi-continuous* at *x_0_* if for every *ε*>*0* there exists a neighbourhood *U* of *x_o_* such that

$$f\left(x\right)\leq f\left(x_{o}\right)+\varepsilon \;\;for\;all\;x\in U\;$$

In the similar way, the *lower semicontinuity* takes place if

$$f\left(x\right)\geq f\left(x_{o}\right)-\varepsilon \;\;for\;all\;x\in U\;$$

**Fig. 1:**
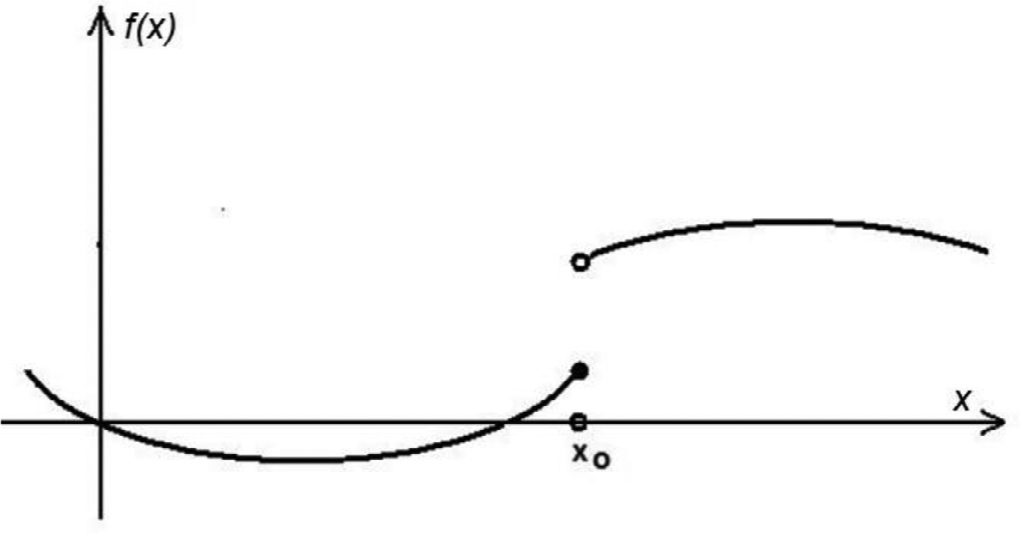
A lower semi-continuous function.

A function is upper- or lower-semi-continuous, if the above condition holds for all *x_o_* in the interval under consideration. Fig. 1 shows an example of a lower semi-continuous function. Note that the function value at *x_o_* is defined as shown by the black dot. Roughly speaking, the function cannot have "jumps" to lower values in any point where it is defined.

The continuity can be defined also for set-valued functions, using the metric induced by the Hausdorff set-to-set distance. As for the lower semi-continuity, the following definition is used.

Let *F* be a mapping from *R^n^* to subsets of *R^n^*. We say that *F* is *lower semi-continuous (l.s.c.)* at x_o_∈ *R^n^* if and only if for any open set *V⊂ R^n^,* such that *F(x_o_)∩V*≠Ø, there exists a neighborhood *U⊂ R^n^* of *x_o_* such that

∀x∈U:F(x)∩V≠Ø;

The mapping *F* is said to be l.s.c. on *R^n^* if *F* is l.s.c. for every *x∈ R^n^.*

The lower semi-continuity is an important property of certain set-valued functions associated with the set-valued function that is used as the right-hand side of the differential inclusion, described further on.

Consider a mapping *F: R^n^* × *I→ P(R^n^)*, where *P(X)* denotes the power set i.e. set of all subsets of a space *X, I* is an interval, *I⊂R.* A *selection* (or *selector*) of *F* over *I* is a function *z(t),* such that *z(t)∈F(x,t)* for all *t∈I*. The existence of selectors is a consequence of the known Axiom of Choice, see Halmos (1960).

Some facts related to selections are quite interesting and may contradict our intuition. For example, this is not true that a continuous field should have a continuous selector. Aubin and Cellina [1] show an example of such field. On the other hand, a discontinuous field may have continuous selections. For example, let the value of *F:[0,2]→R^2^* be a filled rectangle with vertices

(-1,1), (1,1), (1,-1) and (-1,-1) for *t* ≤ 1
and a filled rectangle
(-0.5,0.5, 0.5,0.5, 0.5,-0.5) and (-0.5,0.5) for *t* > 1.
The field is discontinuous at *t* = 1. However, it has a continuous selection *z(t)* ≡ 0.

Let *U* be a compact separable^1^ metric space (in our case a closed and bounded subset *of R^n^*). Assume that there exists an interval *I* and an absolutely continuous function *x(t):I→ R^n^*, such that a.e. in *I, x'(t) ϵ f(x(t),U)* (*x'* stands for *dx/dt*).

Then, there exists a measurable *u: I→U* such that

x(t)=f(x(t),u(t))a.e.inI

(Aubin,Cellina,Chapter 1, Section 14, Corollary 1).

This corollary may be useful while treating with control systems.

Consider a differential equation

$$x\left(t\right)=f\left(x\left(t\right)\right),t),x\left(t_{o}\right)\subset R^{n},t\in I=\left[t_{o},t_{1}\right],t_{o}<t_{1},$$

with Lipschitz-continuous right-hand side. A solution to this equation is a function *x(t)* that is measurable and differentiable almost everywhere and fulfills the equation a.e. over a given time interval *I*.

*Dfferential Inclusion* (DI) is defined by the following statement.

(1) $$\frac{\mathrm{dx}}{\mathrm{dt}}\in F\left(x,t\right),\;x\left(0\right)\in X_{o}$$

where *t* is a real variable (representing the *time* in this article), *x* is a function of time, *x(t)* ∈ *R^n^, F* is a mapping from *R^n^*x*R* to subsets of *R^n^* and *X_0_* ⊂ *R^n^* is the initial set. *R^n^* is the real n-dimensional Euclidean space, and X_o_ is the initial set. In the following, *R* is equal to *R^1^. F* is also called *the set of admissible directions.*

We will call the function *x(t)* a *trajectory* of the DI, if it satisfies (1) a.e. over the interval under consideration. The trajectory must be absolutely continuous and almost everywhere differentiable function.

The *reachable set* of a DI is defined as follows.

First, recall that the *graph* of a function *f(t)* is the set of all ordered pairs (*t, f*(*t*)).

Let *X_o_* be a closed and connected subset of *R^n^, I*⊂*R* denotes an interval *[t_o_,t_1_], x(t)ϵR^n^* is the model state vector*, F: R^n^xR→P(R^n^)* is a set-valued function, where *P(X)* denotes the power set i.e. set of all subsets of a space *X.*

The *reachable* or *attainable set* of (1) is defined as the *union of the graphs of all trajectories* of (1).

The term *emission zone* has been used in the early works. Here, we will rather use the term reachable or attainable set. In many works on the DIs, the mapping *F* is called a field of permissible directions, and a trajectory of the DI is also called a trajectory of the field *F*.

Let us comment on the term "solution to the DI". It is commonly understood that a trajectory of the DI is it's solution. Observe that a DI normally has an infinite number of trajectories. Thus, the trajectory cannot be just called "the solution". Our point is that THE solution to a differential inclusion is given by its reachable set. If we consider a sequence of DIs with shrinking right-hand side that, in the limit, degenerates to a single-valued function, then the corresponding sequence of reachable sets tends to the graph of the solution of the resulting differential equation. This is an argument to call the reachable set the solution to the DI. However, to avoid any ambiguity and conflict of terms, the term "solution to a DI" will not be used in following sections. Instead, we will discuss trajectories and reachable or attainable sets.

An absolutely continuous function *x(t)* is called a *quasitrajectory of the DI* (1) over an interval *I* with initial condition ***x_0_***, if a sequence of absolutely continuous functions *{x_i_}* exists such that

(2) $$\left\{ \begin{array}{c} \left(i\right)\;x_{i}\left(t\right)\to x\left(t\right)\forall \;t\;\in I=\left[t_{o},t_{1}\right]\\ \left(ii\right)\;d(x\left(t\right),F\left(x_{i}\left(t\right),t\right))\to \;0\;almost\;everywhere\;on\;I\\ \left(iii\right)\;x_{i}\left(t\right)\;are\;equibounded\;on\;I\\ \left(iv\right)\;x\left(0\right)=x{}_{o}. \end{array}\right.$$

Recall that a sequence of functions $x_{k}:\left[t_{o},t_{1}\right]\to R^{n},k=1,2,3,...$, *t_o_<t_1_,* is said to be *equibounded* if such *M* exists that

$$|x_{k}\left(t\right)|\leq M\forall k=1,2,3,...\wedge t\in \left[t_{o},t_{1}\right].$$

A function *x = x(t)* is called a *strong quasitrajectory* of the field *F* if there exists a sequence *x_i_(t)* (*i* = 1, 2, ...) of trajectories of *F*, such that *x_i_(t) → x(t).*

Turowicz [22] has given some sufficient conditions for a quasitrajectory to be a strong quasitrajectory. Let us notice that the notion of strong quasitrajectory ("*sliding regime*") was introduced independently and earlier by Filippov (1967) under stronger hypotheses. However, that paper did not refer to the theory of Marchaud-Zaremba.

The set *E* = *conv(F)* is defined as the smallest convex and closed hull of the set *F,* and the set *Q = tend(F)* is the smallest closet subset of *F* that has the same convex hull, *conv(Q) = conv(F).* Note that the *tendor set Q* of Fig. 1.3 contains the curved sections *a* and *b* and the point *c*.

A very useful property of quasitrajectories of the fields *F, E* and *Q* was found by Wazewski. He pointed out that if the field *F* is continuous in the Hausdorff sense, then the field *E* is also continuous and the field *Q* is lower semi-continuous. The most important property of these fields is that, under some additional regularity conditions, **the fields *F, E* and *Q* have the same quasitrajectories**. Moreover, the Filippov-Wazewski theorem states that if *F* satisfies the Lipschitz condition, then for each quasitrajectory, a sequence of trajectories exists that converges to that trajectory. Consequently, the three fields have the same reachable sets with accuracy to their closures (reachable sets of the fields *F* and *Q* need not to be closed). In other words, the sets of trajectories of the inclusions (*x'(t)* stands for *dx(t)/dt*)

(3) $$x^{\prime }\left(t\right)\in F\left(x\left(t\right),t\right)\;\mathrm{and}\;x^{\prime }\left(t\right)\in Q\left(x\left(t\right),t\right)$$

are dense in the set of trajectories of the inclusion

(4) $$x^{\prime }\left(t\right)\in E\left(x\left(t\right),t\right)$$

The word "dense" means that in any neighborhood of a trajectory of the first inclusion there exists a trajectory of the second. This also means that, in many cases of control systems, the "*tendor*" or "*bang-bang*" type of control (field *Q*) can be used without restricting the system reachable set. See also ([18],1986).

Let us denote the set of all trajectories of the field *F* as *{F}* and the set of all its quasitrajectories as {F}*. By *comp (R^n^)* we denote the collection of all nonempty compact subsets of *R^n^.*

Let *T =* (- ∝, + ∝), *W = R^n^* x *T.* Consider the following hypothesis:

**Hypothesis *H(F).*** For *each (x,t)* ***∈*** *W, F(x,t)* ***∈*** *comp (R^n^), F(x,t)* is bounded and continuous on *W*.

Wazewski [23] pointed out that under the Hypothesis *H(F)* we have

(5) {F}*={Q}*={E}*={E}

Let *W = R^n^* *× T* and *Θ(k)* be the hyperplane *t = k.* Also, denote *S(E,k) = {E}* ∩ *Θ(k)* (the "time section of *{E}*). One of the results shown by Zaremba [27] is that *S(E,k)* is a compact and connected set. Thus, the same property holds for the sets of quasitrajectories of the fields *F* and *Q*. A more detailed discussion on this and other properties of the reachable sets can be found in Wazewski [24]. For the field *E* It was also known that if a point in *I × R^n^* is accessible from the initial point *x(0)* of (1), then it is also accessible in optimal (minimal) time.

## Differential inclusions and control systems

The DIs are closely related to control systems. To see this, consider a dynamical system

(6) $$\begin{array}{c} \frac{dx}{dt}=f\left(x\left(t\right),u\left(t\right),\;t\right),x\left(0\right)=x_{o},u\left(t\right)\in C\left(x\left(t\right),t\right)\\ x,u\in R^{n},\;\;C\left(x\left(t\right),t\right)\subset R^{m}\; \end{array},$$

where *x(t)* is the *n*-dimensional system state, *u(t)* is the *m*-dimensional control variable and the set *C* represents the control restrictions. Define a mapping *F* as follows.

F(x,t)={z:z=f(x,u,t):u∈C(x,t)}

Using the set-valued function *F* in the differential inclusion (1), we obtain the DI derived from the control system (6):

(7) $$\frac{dx}{dt}\in F\left(x\left(t\right),t\right),x\left(0\right)\in X_{0}$$

The control system (6) and the DI (7) have the same trajectories. In (7) the control variable does not appear explicitly. In other words, the function f (x,*,t) is a mapping

*f: C → F* for each fixed *x* and *t.*

Define the *bang-bang kernel* of *C(x,t)* as follows.

(8) B(x,t)={u:u∈C(x,t),f(x,u,t)∈Q(x,t)}

The consequence of the equalities (5) is that we can use the bang-bang kernel *B* instead of *C* to obtain a control system with the same quasitrajectories. This means that we can hit or approximate any point inside the reachable set of (7), using the *restricted control set B*. Note that the set *B* contains less points than C. In many practical applications *B* can be reduced to a finite number of points. This permits us to use a simple bang-bang control instead of continuous control, with less complicated instrumentation.

As a consequence of the above remarks, we may conclude that given a differential inclusion, we can find the corresponding control system by parametrizing the function *F* with certain parameter *u* (control variable). This is true in many cases. However, the parametrization problem is not so simple. Consult Aubin and Cellina [1], chapter 1 Section 7, "Application: The parametrization problem". In that section, it is pointed out that the existence of continuous selection of *F* is not sufficient to enable parametrization of *F*. Fortunately, the mappings considered in the following are regular enough to permit parametrization.

## Differential inclusion solver

As stated before, the original algorithm of the solver has been published in Raczynski [17]. Here we present the new version that includes the use of multiprocessing, improved accuracy and graphical presentation of the results.

The algorithm of the DI solver has been coded using the Embarcadero® Delphi, and requires that package to be installed on the user's machine. A limited, stand-alone ".exe" version of the solver is also available. Our main goal is the RS determination and not optimization. The DI solver and the present problem statement should not be confused with the *differential inclusions method* used in the optimal control problems.

We are looking for the reachable set for a given initial condition or initial set. It is known that, with a sufficient regularity assumptions, the reachable set is continuous with respect to the initial and it is connected for any fixed time instant. The reachable set need not be convex and may have rather complicated shape. It might appear that a simple way to get the shape of the reachable set is to calculate a number of solutions equation

$$\frac{dx}{dt}=z\left(t\right),$$

for different functions *z(t),* where the functions *z(t)* are *selectors* of the DI (1), It might appear that choosing *z(t)* randomly*,* we can cover the inside of the reachable set with sufficient density and then estimate its shape. Unfortunately, **this is not true**, even if we select only *z(t)* belonging to the boundary of *F*. We will call this method *simple or primitive shooting* and compare it with the DI solver algorithm. A simple simulation shows that even in very simple cases the set of trajectories provided by primitive shooting (using any density function) is concentrated in some small region inside the reachable set and does not approach its boundary. The problem is that the resulting set of trajectories has a very narrow distribution inside the reachable set, even if the distribution used to generate selectors is not narrow, for example uniform inside *F*.

In the present DI solver, we explore the **boundary** of the reachable set, and not of its interior. With enough trajectories that belong to the boundary, the shape of RS can be estimated with reasonable accuracy. The problem is to generate these trajectories in such a way that the density of points be nearly uniform on the boundary. This permits to avoid "holes" in the resulting graphical image.

**Fig. 2:**
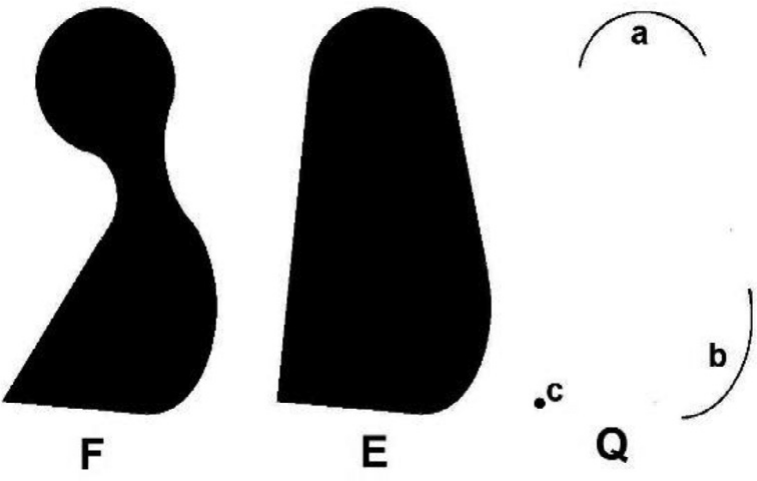
Example of the sets F, E and Q.

To generate such "boundary trajectories" we use well known methods of the control theory. If the field *F* is not convex, but Lipschitzian, it is sufficient to estimate the reachable set for trajectories of the corresponding tendor (see Section 2) field instead of the original field *F*. This can be easier because the tendor field contains few points, in many cases only isolated extremal points of the given set *F*. Recall that the fields *F, E*, and *Q* (Fig. 2) have the same quasitrajectories and that for each quasitrajectory a regular trajectory exists nearby.

The Maximum Principle (Pontryagin,1962) states that the necessary condition for a trajectory to be optimal, is to maximize the expression called Hamiltonian for each time instant over the time interval under consideration (see chapter 2). In other words, the Principle permits us to decompose the original optimization problem of maximization of a **functional** into a se of problems of **function** maximization. The original optimization problem is as follows. Given a control system described by the Eq. (6), we look for an optimal control and the corresponding optimal trajectory that minimizes a given criterion (9) over the interval *I = [0,T]*.

(9) $$\min J=\int_{0}^{T}f_{0}\left(t\right)dt$$

If *f_o_*≡1, then the trajectory is time-optimal, i.e. reaches the final point in optimal time.

To define the Hamiltonian, we must define the conjugated vector *p є R^n^* that satisfies (by definition) the following equations.

(10) $${dp}_{i}/dt=-\sum_{j=1}^{n}\frac{\partial f_{j}}{\partial x_{i}}p_{j}-\frac{\partial f_{0}}{\partial x_{i}},$$

where *i = 1,..,n* and *f* is the vector of the right-hand sides of (6). Observe that the necessary condition for a trajectory to be time-optimal, is to entirely lay on the boundary of the reachable set. This means that we can suppose *f_o_*≡1, and eliminate *f_o_* from the above equations.

The Hamiltonian function is defined as follows.

$$H=\sum_{j=1}^{n}p_{j}\;f_{j}$$

The Maximum Principle requires that the necessary condition for the trajectory to terminate at a boundary point of the reachable set is that the control *u(t)* maximizes the Hamiltonian over the interval *I.* This can be used to generate boundary trajectories of the differential inclusion. If the inclusion is given in the form of a control system (6), then we apply the Principle directly. If it is given in the general form (1), we must parametrize the set *F* and treat the parameter as the control.

The vector *p* must satisfy the *transversality conditions* [9]. This provides the final condition for the conjugated vector. Consequently, to calculate an optimal trajectory with a given optimality criterion, the *two-point boundary value problem* must be solved The boundary conditions for the state vector are given at the initial time, and the boundary conditions for the conjugated vector are known at the end of the trajectory.

In our case we do not need to solve the two-point boundary condition problem. Observe that starting with **any** initial condition for the conjugated vector and maximizing the Hamiltonian on each time step, a single **forward** integration of the Eq. (6) and (10) provides a trajectory that lies on the boundary of the reachable set. Thus, we can choose the initial conjugate vector randomly, obtaining random final boundary points (and not the points inside the reachable set). We do not solve any particular optimization problem, but we are just looking for the trajectories that scan the boundary of the reachable set. The problem is how to generate the initial vector *p* to cover the resulting final boundary set with uniform density of points and to avoid holes in it.

The distribution *D* used below is a probability distribution function defined inside the n-dimensional unit cube with center at the origin of the coordinate system. The algorithm is as follows (the discrete-time version of the Maximum Principle is used).

The new, parallel version of the DI Solver algorithm is as follows

0. *Define D as the uniform density function, set x = x_0_ .*
1. *Generate initial vector p according to the density D.*
3. *Launch several concurrent tasks on the available processors of the current machine, each of them, integrating the*Eqs. (6)*and (*10*) over the interval I. In each trajectory use the control u that maximizes the Hamiltonian at each integration step.*
4. *In each integration task, store the initial p and the whole trajectory in a consecutive record of a file.*
5. *Select the final point x_k_ that lies in a region of minimal density of points x, searching in the file where trajectories have been stored.*
6. *Modify the distribution D, increasing the probability density in a neighborhood of the point p_k_ that corresponds to the point x_k_.*
7. *If there are enough trajectories stored, then stop, otherwise go to Step 1.*

Note the difference between the original version of the solver and the present one. The trajectory integration is now executed concurrently on multiple CPU processors, making the algorithm several times fasted. By the term "region of minimal density of points" (point 5) we understand the spot of low-density of points on the image of the final reachable set or its two-dimensional projection. This part of the algorithm is rather heuristic.

It is important to notice that, although we randomly generate the initial conditions for the conjugated vector, this algorithm has nothing to do with the *simple shooting* method, mentioned earlier. We do not explore the interior of the RS, but scan its boundary. In fact, the random generation of initial vector *p* can be replaced by other, deterministic, method of search.

The stop condition may rather be based on the user decision. As the result, we obtain a 2D image that is a projection of the reachable set on a given plane. In the case of a second order system this image should be a closed curve, for systems of higher order it will be a cloud of points in *R^n^*. The user can stop the program if he can recognize the shape of the reachable set, and identify its boundary. Practical experiments show that this can be reached after integrating 500 -1000 system trajectories. Anyway, this procedure may be difficult to use for models of higher order (more than 10, perhaps).

The maximization of the Hamiltonian (Step 3) can be done using any maximization procedure.. This procedure is not predefined because it may depend on each particular case. Note that here we reduce the original problem of solving a DI to some "sub-problems" that may not be easy to solve, but belong to well-known fields of optimization techniques. When the original system is linear with respect to the control vector and the restriction set is a multidimensional cube, then the maximization can be reduced to a simple scan over a finite number of points. We will not discuss here the methods of maximization of the Hamiltonian (Step 3). There exists a huge literature on it in the field of control theory [9], [15].

The program also contains other mechanism that helps us obtain more uniform point distribution. This is a simple scanning that eliminates points which lie very close to each other or are identical. Such double points can appear because the system we analyze is a discrete-time approximation of the original continuous system. The effects of time-discretization can be seen on the obtained images.

An important question is if, and when, you really need the reachable set calculated by the DI solver. Obviously, if our system is of the first order, the determination of the reachable set is rather trivial and can be done easily without involving DIs. In some cases, when the modeled system is of higher order, but strongly damped, the extreme points of the RS can be calculated simply by applying the extreme values of control variables. However, in a general multidimensional case (even if the system is of the second order and linear), the constant extreme controls (extreme points of the control restriction set) do not correspond to the extreme or boundary points of the system reachable set. The RS becomes a complex multidimensional shape, not necessarily convex, with the boundary surface that may fold several times. Even in a simple two-dimensional case, the mapping from the permitted control set *C* to the RS is highly irregular.

In some situations, the DI solver may fail. This occurs when the analyzed model includes stiff equations. This is normal, recall that most of the numerical methods for ordinary differential equations also fail for such type of equations, and the treatment of stiff systems requires special algorithms. The model stiffness is not always easily detected. The solver failures may, for example, occur when the model if of order four or higher, and includes parts that oscillate at high frequency. For example, the model of quarter-car suspension has the proper ("slow") oscillations of the spring-damper-mass part, and the stiff part that takes into account the tire elasticity and the mass of the wheel.

There are several improvements in the new version of the DI solver. The algorithm that modifies the density *D* has been revised and improved, to avoid possible "holes" in the images of the RS. Also the display of the image is considerable improved, and permits easily rotate the 3D RS image to better understand the shape of the set. It is very important for models of dimensionality greater than two. For such models, the time-section of the reachable set cannot be seen as a well-defined contour. For example, if the model is composed by the set of three equations, the final RS is a cloud of points distributed over a 3D surface, like an air balloon. If we look at a 2D projection of this object, it might appear that some points lie inside the reachable set, what is not true. Rotating the image gives us a better understanding of the spatial distribution of points.

The algorithm was adopted for multi-processing. Note that the operations of integrating model trajectories can be executed concurrently. This accelerates the task due to the number of processors. Running on a quad CPU we complete the task four-times faster. Further improvements can be done using the GPU (Graphical Processing Unit) that may contain hundreds of processors. In this case, we could reach the velocity of RS calculation comparable with the speed of recent numerical methods for the ordinary differential equations. However, such implementations are always hardware-dependent, and the practical applications are available mainly with the NVIDIA graphic cards.

As mentioned before, the DI solver runs over the Delphi package. The main algorithm is coded in the form of a Delphi unit. The right-hand-side expressions for the model equations are defined by the user for each particular case. This means, that these expressions must be compiled to be executed. Consequently, the algorithm must invoke a corresponding compiler, in this case the Delphi compiler. The new, stand-alone exe version of the solver has been developed, that does not require Delphi. This version has its own compiler for mathematical expressions. However, this compiler is rather slow compared that of Delphi, and does not use multiprocessing. This version of the solver can be used for simple examples and not very complicated models.

In the following section we can find an example of reachable set images produced by the DI solver, applied to the problem of the *functional sensitivity*.

## Functional sensitivity

The classical, local sensitivity analysis (basic local version) uses the partial derivatives of the model output *Y*, with respect to components of an input vector (model parameters) *u=(u_1_,u_2_,...,u_n_),* at a given point *u_0_:*

(11) $${\left|\frac{\partial Y}{\partial u_{i}}\right|}_{u_{0}}$$

The derivative is taken at some fixed point in the space of the input (hence the 'local' in the name of the analysis mode). The use of partial derivatives suggests that we consider small perturbations of the input vector, around the point of interest *u_0_*. Consult Cacuci. There are several kinds of sensitivity analysis. Consult, for example, *scatter plots,* Friendly and Dennis [7], r*egression analysis* Freedman [6] or Cook *variance-based model* or Sobol methods, Sobol [19], the *screening method,* Morris [12] or *logarithmic gain,* Sriyudthsak et al. [20].

The System Dynamics software offers tools for *dynamic sensitivity analysis*. Programs like Vensim or PowesSim include procedures that generate multiple model trajectories where the selected model parameters vary from one trajectory to another. However, in these packages the parameters are constant along the trajectory. Our approach is different. As explained in the following sections, we treat the perturbations as functions of time. The main tool used here is the differential inclusion.

Consider a dynamic model described by an ordinary differential equation

(12) $$\frac{dx}{dt}=f\left(x,u,t\right)\;,$$

where *x = (x_1_,x_2_, , ,x_n_)* is the state vector, *u = (u_1_,u_2_,...,u_m_)* is the perturbation (parameters, control) vector, *t* is the time. We have *x ϵ X, u ϵ U, f:X*x*U*x*R→X*. Here, *X* is the state space, *U* is the control space and *R* is the real number space. We restrict the considerations to the case *X = R^n^, U = R^m^, R = R^1^, R^k^* being the real Euclidean k-dimensional space. Let *t ϵ I=[0,T],* and *G* be the space of all measurable functions *u: I→R^m^.*

Now, consider a variation *δu* of *u* and a perturbed control *u'*. The variation is a function of time, so that *u'(t) = u(t)+ δu(t)* for all *t* ϵ *I.* The solution to (12) over *I,* with given initial condition *x=x_0_* and given function u(*) will be called a trajectory of (12). Thus, any component *x_k_* of the final value of *x(t)* depends on the shape of the whole function u(*). In other words*, x_k_ (t) = x_k_(t)[u']* (for any fixed *t*) is a *functional* (not a function) of u'(*). Unlike a function, in our case, a functional is a mapping from the space *G* to *R*. Denote *δx_k_=x_k_[u+ δu]-x_k_[u]= x_k_[u']-x_k_[u].*

In this book, the *local functional sensitivity* is defined as

(13) $$S_{k}=\left|\frac{\delta x_{k}}{\delta u_{0}}\right|$$

Note the difference between the conventional local sensitivity (11) and the functional sensitivity (12). The notation *δu* is nothing new, it denotes the variation of the function *u*, as defined in the calculus of variations (see [4], [13]).

The term (13) defines a local property of the trajectory *x_k_(t).* Here, we are interested rather in the response of the model to perturbations that are not necessarily small. We will not enter in the methodology of the variational calculus. Our task is to define the *functional sensitivity* as the set of the graphs of all trajectories of (12), where *u = u_0_+Δu.* Here, *Δu(t)* is a limited perturbation, not necessarily small. This is equivalent to say that *u(t)* belongs to a set of restrictions *C(t), u(t) ϵ C(t),* for all *t ϵ I*. Here, *C(t)* is a subset of *R^m^.* When *u* scans all possible values inside the set *C*, then the right-hand side of (1.10) defines a set-valued function. This way, (12) with disturbed control also defines the corresponding differential inclusion.

The functional sensitivity defined this way is non-local. We do not use the term "global", because this is not a global property of the model. We only do not require the perturbation to be small.

**An example.**

Consider a simple non-linear model of the second order:

(14) $$\left\{ \begin{array}{c} \frac{dx_{1}}{dt}=x_{2}\\ \frac{dx_{2}}{dt}=1-0.2u_{1}-x_{1}-0.1u_{2}\left(x_{2}+2.3x{}_{2}^{2}\right) \end{array}\right.$$

where *u_1_* and *u_2_* are uncertain parameters. Let the parameter *u_1_* fluctuate between -0.2 and +0.2, and parameter *u_2_* fluctuate between 0.025 and 0.175. The initial conditions are *x_1_=x_2_*=0, and the final simulation time is equal to 10.

**Fig. 3:**
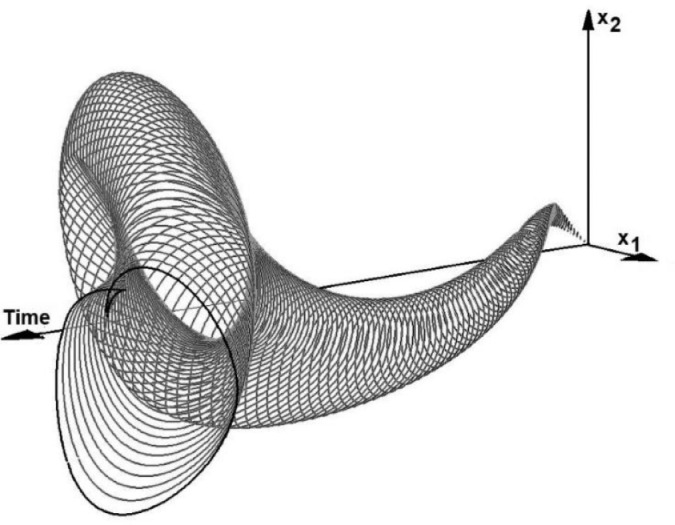
Functional sensitivity of model (14). The image of the reachable set produced by the DI solver.

Fig. 3 shows the 3D image of the model RS. The three axes of the plot represent *x_1_, x_2_* and the time. The image was generated by the DI solver, described in chapter 3.

It can be seen that even for relatively small perturbation *u_1_* and *u_2_,* the deviation of the state vector may be quite big.

**Fig. 4:**
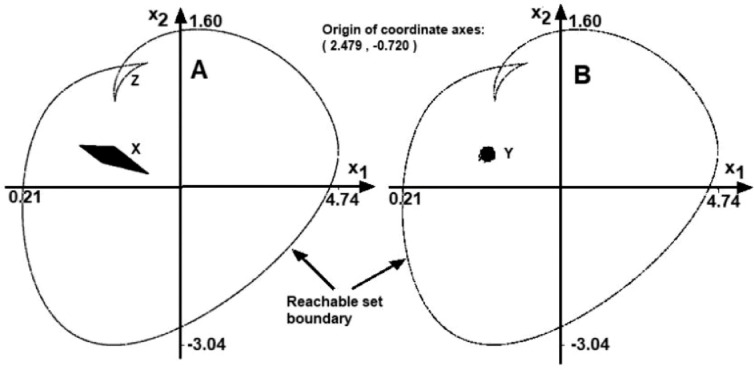
Final contour of the reachable set. Comparison with the simple shooting.

Fig. 4-A depicts the comparison of the functional sensitivity to the conventional sensitivity analysis provided in some system dynamics packages. The contour indicates the boundary of the reachable set for the model (14) at *t* = 10. These are end points of about 2000 boundary-scanning trajectories. A small black region marked with X is the result of the "Vensim-like" sensitivity analysis, where the parameters are constant along each trajectory. The region X was obtained by generating 50000 trajectories with the same limits of uncertain parameters. At the part B of the figure we can see the region Y, obtained by generating 50000 trajectories where the parameters can change the value randomly, within the same limits at each integration step.

Note a small part of the contour marked with Z. These are points generated by the solver that belong to the interior of the reachable set. Recall that the Maximum Principle provides the necessary and not sufficient conditions for the trajectory to be optimal. Consequently, when the reachable set is more complicated and folds several times, we can obtain also such extra points.

**Fig. 5:**
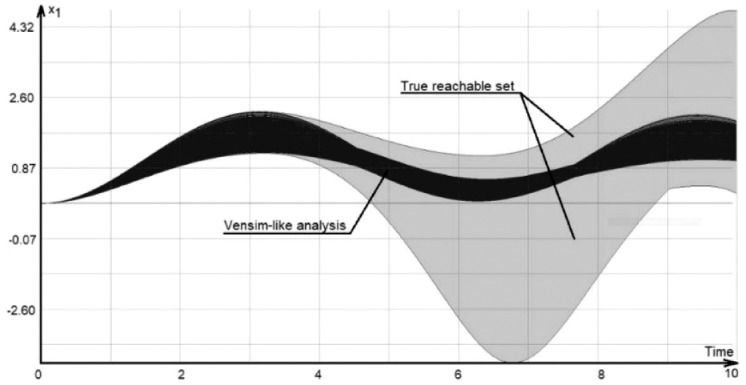
Functional sensitivity set projected into the *x_1_-time* plane.

Fig. 5 shows a side view of the same reachable set produced by the DI solver, projected into the *x_1_-time* plane. Note that the functional analysis region coincides with the conventional results for the initial time interval [0,35], but it is very different from the true reachable set for greater time interval.

To perform the functional sensitivity analysis, the DI solver must be used. It provides the estimates of the reachable sets. Comparing functional sensitivity with the conventional sensitivity analysis we can see that the obtained reachable sets almost always several times greater than that obtained using the classical approach.

## Other applications

The RS calculation is an important tool for treatment of uncertainty in dynamic systems. Note that uncertain parameter should not be confused with a random-valued parameter. The uncertain variables need not be random, neither have any probabilistic properties. They are *tychastic* variables, as defined in Aubin and Saint-Pierre [2]. The reachable set shows the influence of such uncertain variables on the system behavior.

### Mechanical system

**Fig. 6:**
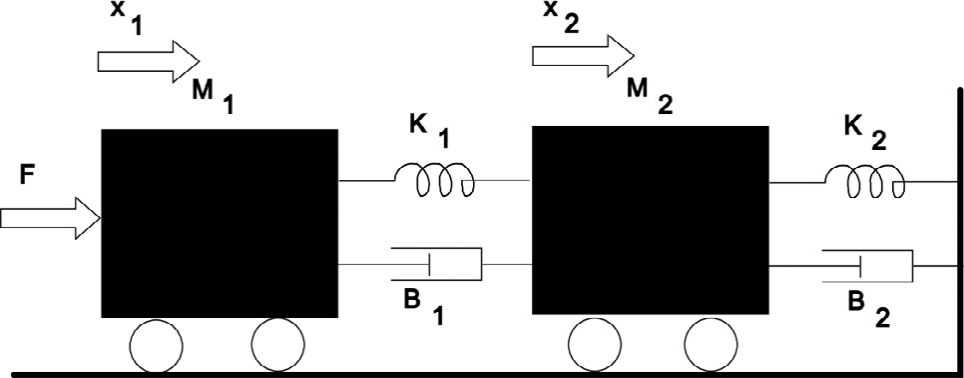
Example of a mechanical system.

A simple mechanical system is shown in Fig. 6. The system equations are as follows.

$$\begin{array}{c} x_{1}=x_{3}\\ x_{2}=x_{4}\\ x_{3}=\frac{1}{M_{1}}\left[F-K_{1}\left(x_{1}-x_{2}\right)-B_{1}f\left(x_{3}-x_{4}\right)\right]\\ x_{4}=\frac{1}{M_{2}}\left[K_{1}\left(x_{1}-x_{2}\right)+B_{1}f\left(x_{3}-x_{4}\right)-K_{2}x_{2}-B_{2}f\left(x_{4}\right)\right]\\ where\;f\left(x\right)=x^{2}\;sign\;x \end{array}$$

**Fig. 7:**
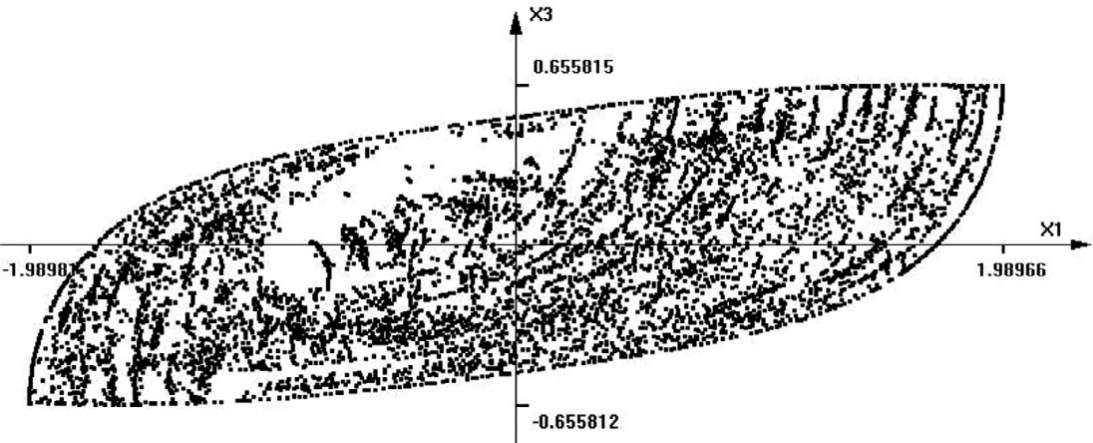
The shape of a time-section of the reachable set of a 4-th order system (Example 3).

Note that the dumpers are non-linear. *F* is an external force that belongs to *[-0.5, 0.5].* The time-section of the reachable set for this system is shown in Fig. 7. Here, *time = 4, m1 = 1, m2 = 2, k1 = 0.3, k2 = 0.1, b1 = 1.5, b2 = 3.0* . Note that some points appear to belong to the interior of the set. Those are not the points obtained by primitive shooting, as in the previous example. In fact, all these points belong to the boundary of the reachable set. What we see is only a projection of a 4-dimensional figure (point cloud) onto a 2-dimensional plane *x_1_, x_2_* for a given time instant.

**Fig. 8:**
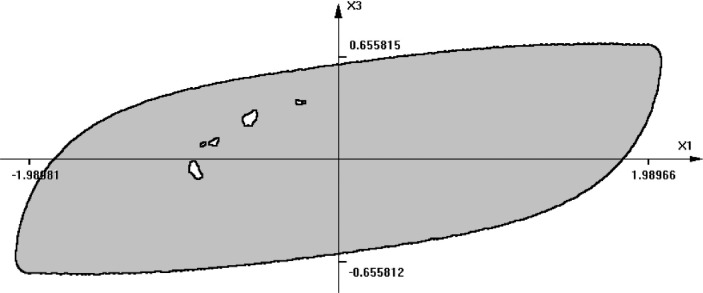
The shape of the reachable set of Fig. 7 enhanced by fuzzy sets technique.

The graphical representation of the reachable sets for models of dimensionality greater than 3 is somewhat difficult. The question is how to display a cloud of points of n-dimensional space in order to show clearly the shape. If the cloud is 3-dimensional, this can be done by displaying a rotating 3D image to produce an illusion of 3D viewing. Other possible enhancements may be obtained using techniques known in fuzzy set theory. Fig. 8 shows the result of calculating the fuzzy variable representing the level of membership in the region. Points with membership value greater than 0.5, are shown as gray pixels. If there are not enough points to analyze, then the holes in the region may appear. Anyway, such images always depict approximate shapes.

### Example 5: Lotka-Volterra equation

Lotka-Volterra (L-V) equations describe the dynamics of prey-predator ecological systems (Takeuchi 1996).

In the simplest case of two species, the prey population (for example rabbits) grows due to the birth-and-death process. The growth would be exponential, but it is a limitation: there is a predator (e.g. wolves) who eat rabbits. The population of wolves grow when they have food, but if there are few rabbits available, the wolves die. Denote the rabbit population size as *x_1_* and the wolves as *x_2_*. The classical form of two-species Lotka-Volterra equations is as follows.

$$\begin{array}{c} \frac{dx_{1}}{dt}=ax_{1}-bx_{1}x_{2}\\ \frac{dx_{2}}{dt}=-cx_{2}+dx_{1}x_{2} \end{array}$$

In the first equation, the term *bx_1_x_2_* means that the rate of rabbits caught by wolves is proportional both to wolves number and rabbits number. A similar term appears in the second equation, telling that the growth rate of wolves increases when they have more food. Coefficient *a* defines the rabbits natural grows rate, and *c* defines the wolves natural death rate. There are many other versions of the equations used in ecological models, with two or more species in the N-species food chains.

Now, consider the two-species system with some uncertainty. Namely, suppose that the growth rate of the rabbits is uncertain, subject to climate changes and other external factors. For example, suppose that the parameter a may change in time, within the range of +-25%. Thus, the system equation can be written as follows.

$$\begin{array}{c} \frac{dx_{1}}{dt}=a\left(1+u\right)x_{1}-bx_{1}x_{2}\\ \frac{dx_{2}}{dt}=-cx_{2}+dx_{1}x_{2} \end{array}$$

Where *u* changes between -0.25 and +0.25. This way we obtain a differential inclusion, with the right-hand side parametrized by the variable u.

**Fig. 9:**
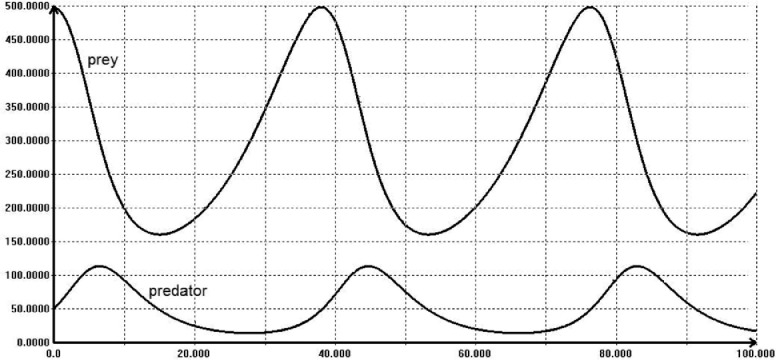
A simple simulation of the Lotka-Volterra model.

A simple simulation of the above equation, with u ≡ 0 is shown on Fig. 9.

The L-V equations have strong nonlinearities (products of two state variables). Thus, the solution reveals a series of non-sinusoidal oscillations. The model parameters are: *a* = 0.1, *b* = 0.02, *c* = 0.3, *d* = 0.001 and final simulation time equal to 100.

**Fig. 10:**
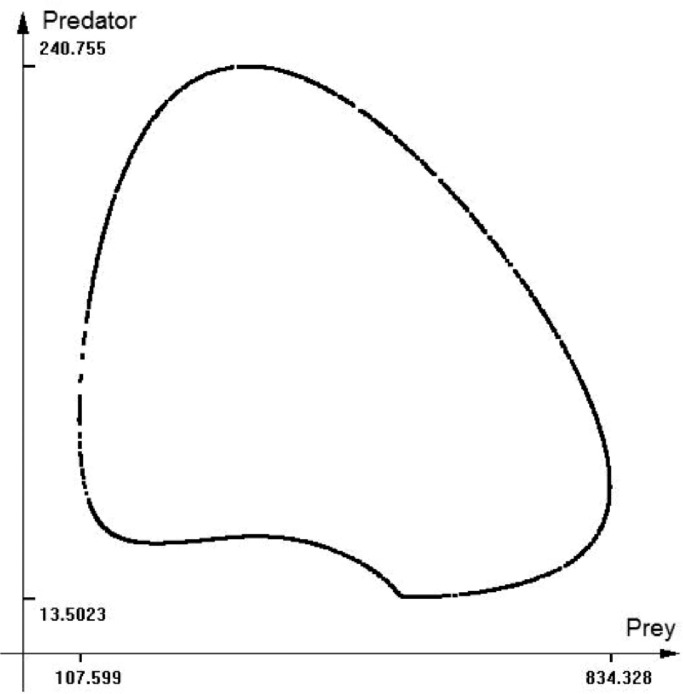
The time-section of the reachable se for the L-V equations. Time = 45.

**Fig. 11:**
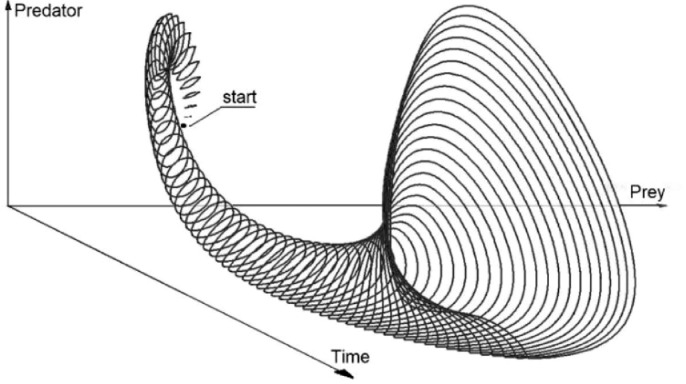
The shape of the reachable se of the L-V equation. 3D image. time equal to 45.

Now, applying the DI solver we obtain the attainable set of the size of the two species in presence of uncertainty. Fig. 10 shows the shape of the reachable set boundary for time = 45. In Fig. 11 we can see the 3D image of the set.

In ecology and population growth models, we almost always have uncertain factors that have unknown probability distribution and stochastic parameters. In these cases, the differential inclusions may be a useful research tool. Observe that for the Lotka-Volterra model even with small fluctuations of uncertain parameter, the size of the reachable set after time approximately equal to the model oscillation period is quite big. This means that this model is useless for the predictions, even for small time intervals.

## Conclusion

New DI solver contains considerable improvements compared to the original version. Running on multiple processors, it can calculate the shape of the reachable set in several seconds. A possible application of the GPU hardware can accelerate the solution hundreds of times. The graphical result display was redesigned to provide a more clear and easier to interpret images. An application to the functional sensitivity analysis shows that, in fact, the RS for dynamic systems is, at the same time, the sensitivity set. This can be applied in the analysis and design of robust control systems.

## Footnotes

1 A separable space is a space that contains a dense enumerable set.
